# PPARγ Antagonists Exhibit Antitumor Effects by Regulating Ferroptosis and Disulfidptosis

**DOI:** 10.3390/biom14050596

**Published:** 2024-05-18

**Authors:** Shiyu Zhang, Ying Wang, Junjie Gu, Yang Yang, Jing Liang, Yimei Wang, Ning Ji, Ming Liu, Yingxin Zhang, Silu Sun, Qianming Chen, Jing Li

**Affiliations:** State Key Laboratory of Oral Diseases, National Clinical Research Center for Oral Diseases, Research Unit of Oral Carcinogenesis and Management, Chinese Academy of Medical Sciences, West China Hospital of Stomatology, Sichuan University, Chengdu 610041, China; zsy799786449@gmail.com (S.Z.); wangying5@stu.scu.edu.cn (Y.W.); jackiegu2019@stu.scu.edu.cn (J.G.); yangyang.roscee@gmail.com (Y.Y.); zhangjane334@gmail.com (J.L.); 2022224030002@stu.scu.edu.cn (Y.W.); jining_1023@scu.edu.cn (N.J.); liuming34@stu.scu.edu.cn (M.L.); 2023224030014@stu.scu.edu.cn (Y.Z.); silusun@scu.edu.cn (S.S.); qmchen@scu.edu.cn (Q.C.)

**Keywords:** OSCC, ferroptosis, disulfidptosis, HMOX1, SLC7A11, dendritic cells, T cells

## Abstract

Oral squamous cell carcinoma (OSCC) stands as a prevalent subtype of head and neck squamous cell carcinoma, leading to disease recurrence and low survival rates. PPARγ, a ligand-dependent nuclear transcription factor, holds significance in tumor development. However, the role of PPARγ in the development of OSCC has not been fully elucidated. Through transcriptome sequencing analysis, we discovered a notable enrichment of ferroptosis-related molecules upon treatment with PPARγ antagonist. We subsequently confirmed the occurrence of ferroptosis through transmission electron microscopy, iron detection, etc. Notably, ferroptosis inhibitors could not completely rescue the cell death caused by PPARγ inhibitors, and the rescue effect was the greatest when disulfidptosis and ferroptosis inhibitors coexisted. We confirmed that the disulfidptosis phenotype indeed existed. Mechanistically, through qPCR and Western blotting, we observed that the inhibition of PPARγ resulted in the upregulation of heme oxygenase 1 (HMOX1), thereby promoting ferroptosis, while solute carrier family 7 member 11 (SLC7A11) was also upregulated to promote disulfidptosis in OSCC. Finally, a flow cytometry analysis of flight and multiplex immunohistochemical staining was used to characterize the immune status of PPARγ antagonist-treated OSCC tissues in a mouse tongue orthotopic transplantation tumor model, and the results showed that the inhibition of PPARγ led to ferroptosis and disulfidptosis, promoted the aggregation of cDCs and CD8^+^ T cells, and inhibited the progression of OSCC. Overall, our findings reveal that PPARγ plays a key role in regulating cell death in OSCC and that targeting PPARγ may be a potential therapeutic approach for OSCC.

## 1. Introduction

Oral squamous cell carcinoma (OSCC) is an aggressive tumor originating from the squamous epithelial cells of the oral mucosa, ranking among the most prevalent cancers affecting the oral cavity. In the majority of cases, surgical intervention is the primary treatment approach for OSCC [1]. The insufficient removal of tumor cells heightens the risk of local and regional recurrence, consequently diminishing long-term survival rates. In the cases of advanced progression, postoperative treatments such as radiotherapy, chemoradiotherapy, targeted therapy focused on oncogenes, and immunotherapy may be administered, but these treatments have not been able to effectively improve the survival rate [2,3]. Hence, the quest for novel treatment modalities for OSCC assumes paramount importance.

PPARγ is a nuclear receptor protein that plays a crucial role in regulating various physiological processes in the body [4]. Several studies have demonstrated that the activation of PPARγ can regulate the cellular uptake and utilization of glucose [5,6]. Notably, the role of PPARγ in tumors is complex, and its outcome appears to be influenced by factors such as cancer type, the stage of development, and specific genetic and molecular background [7]. Previous studies have shown that patients with OSCC exhibiting high levels of PPARγ expression tend to have a less favorable prognosis than those with lower PPARγ expression [8]. However, its regulatory mechanism and targeted therapeutic potential remain unclear. In addition, PPARγ is believed to regulate the intracellular redox balance and lipid metabolism through multiple pathways related to the occurrence and resistance of ferroptosis. PPARγ activation can inhibit ferroptosis by regulating the intracellular antioxidant defense system and reducing lipid peroxidation and the accumulation of iron ions [9,10]. In recent years, ferroptosis has aroused great interest in the cancer community, partially because ferroptosis is a programmed cell death mechanism fuelled by iron-dependent lipid peroxidation, which is different from other forms of regulated cell death in terms of mechanism and morphological characteristics and has enormous potential for treating cancer. Tumor cells have evolved multiple mechanisms to evade ferroptosis and promote tumor development and metastasis, including limiting the accumulation of polyunsaturated fatty acid (PUFA)-PLs and peroxidation, limiting the availability of free iron, and upregulating cellular antioxidant defense system pathways [11,12].

Cystine uptake mediated by SLC7A11 is known to be crucial in shielding cells from ferroptosis [13,14]. Paradoxically, in the instances of glucose deprivation, SLC7A11 promotes cell death [15,16]. Gan et al. revealed that in cancer cells with high SLC7A11 expression, an insufficient NADPH supply caused disulfide stress, which was named disulfidptosis [17]. However, whether disulfidptosis and ferroptosis can coexist in the process of tumor cell death remains to be verified.

In this study, we observed that OSCC cells exhibited a ferroptotic phenotype upon PPARγ inhibition, which could be partially alleviated by ferroptosis inhibitors. Moreover, a disulfidptosis phenotype also developed after PPARγ was inhibited, and this cell death could be improved by simultaneously administering ferroptosis and disulfidptosis inhibitors. Mechanistically, we found that inhibiting PPARγ can promote ferroptosis in OSCC cells by upregulating heme oxygenase 1 (HMOX1) and promote disulfidptosis by upregulating SLC7A11. In a mouse orthotopic transplantation tumor model, after PPARγ was inhibited, the immune cell population increased; these cells included both dendritic cells and CD8^+^ T cells and were recruited through ferroptosis and disulfidptosis, leading to the inhibition of tumor development in OSCC.

## 2. Materials and Methods

### 2.1. Animals

We selected 5- to 6-week-old SPF C57BL/6 adult female mice (weighing 20–25 g) from the West China Center for Orofacial Diseases Model. The mice were given unrestricted access to food and water, placed in pathogen-free cages, subjected to a 12 h light/dark cycle, and kept at a regulated temperature between 20 and 26 °C. The animal experiments were randomized and blinded. The mice were euthanized to minimize their pain as much as possible. The study was conducted according to the guidelines of the Declaration of Helsinki, and approved by the Institutional Ethics Committee of the West China Dental Ethics Committee of Sichuan University (WCHSIRB-D-2023-638, 23 November 2023).

### 2.2. In Vivo Mouse Models and Analysis

4MOSC2 cells were cultured in KSFM, with or without the supplementation of GW9662 (20 µM, Sigma, St. Louis, MO, USA), for a duration of 24 h. Following this, the cells were resuspended in a serum-free medium in preparation for injection. Using sterile disposable 1 mL syringes, the cell suspension, containing 1 × 10^6^ cells in 100 µL volume, was injected into the tongues of the mice. The mice were then divided into two groups: an experimental group and a control group. The experimental group received intraperitoneal injections of GW9662 (2.5 mg/kg) every three days, while the control group was administered DMSO at the same intervals. After 12 days, or when the animals reached euthanasia criteria, they were euthanized. The collected samples underwent comprehensive evaluation using cytometry by time-of-flight (cyTOF) analysis and immunohistochemistry.

### 2.3. Cell Cultures

The human OSCC cell lines, UM1 and CAL27, along with the murine OSCC cell line, 4MOSC2, were kindly provided by the State Key Laboratory of Stomatology, West China School of Stomatology, Sichuan University. The UM1 and CAL27 cells were cultured suitably in Dulbecco’s Modified Eagle Medium (DMEM; Sigma, St. Louis, MO, USA) supplemented with 10% fetal bovine serum (FBS; BI, Kibbutz Beit HaEmek, Israel) and 1% penicillin–streptomycin solution (HyClone, Logan, UT, USA). Meanwhile, the 4MOSC2 cells were maintained in a specified keratinocyte serum-free medium (KSFM; Thermo Fisher Scientific, Waltham, MA, USA). All the cells were incubated at 37 °C in a humidified atmosphere containing 5% CO_2_. The cells were pre-incubated with GW9662 (20 µM), T0070907 (20 µM, Selleck, Shanghai, China), Liproxstatin-1 (2 µM, Sigma, St. Louis, MO, USA), Rosiglitazone (10 µM, Sigma, St. Louis, MO, USA), or TCEP (1 mM, Selleck, Shanghai, China) for 24 h, 48 h, or 72 h, depending on the stimulation conditions. The final concentration of dimethyl sulfoxide (DMSO) used as a solvent did not exceed 0.1%. 

### 2.4. Transmission Electron Microscopy

The UM1 and CAL27 cells (1 × 10^5^) were cultured in either DMEM or DMEM containing GW9662. After incubation at 4 °C, the cells were fixed in 3% glutaraldehyde for 12 h and subsequently in 1% osmium tetroxide for an hour. Following gradual dehydration, the cells were immersed in butyl acetate and then embedded in Epon812 resin. Ultrathin sections approximately 60–90 nanometers thick were cut using an ultramicrotome. These sections were stained with uranyl acetate for 10–15 min and lead citrate for 1–2 min. The images of the copper grids carrying the sections were acquired using a transmission electron microscope (JEM-1400FLASH, JEOL, Tokyo, Japan).

### 2.5. Light Microscopic Study

A TUNEL assay (BOSTER, Wuhan, China) was used to detect apoptosis in the OSCC cells. CM-H2DCFDA (Thermo Fisher Scientific, Waltham, MA, USA) was utilized for ROS detection in the OSCC cells. An Image-iT™ Lipid Peroxidation Kit (Th Thermo Fisher Scientific, Waltham, MA, USA) was used to assess lipid peroxidation. The OSCC tissues were fixed in a 4% neutral-buffered formaldehyde solution (Biosharp, Hefei, China), embedded in paraffin, and sectioned. Histological analysis was performed using hematoxylin and eosin (H&E) staining (Biosharp, Hefei, China). For immunohistochemical staining, antigen retrieval was conducted using either sodium citrate antigen retrieval solution (Beyotime, Shanghai, China) or EDTA antigen retrieval solution (Beyotime, Shanghai, China). The slides were incubated overnight at 4 °C, after which the primary antibodies were diluted with a 3% BSA solution. Antibodies against PPARγ (Abcam, Boston, MA, USA, 1:250), GPX4 (Abcam, Boston, MA, USA, 1:300), SLC7A11 (Abcam, Boston, MA, USA, 1:300), HMOX1 (Abcam, Boston, MA, USA, 1:100), and p-NRF2 (Proteintech, Wuhan, China, 1:200) were used for immunostaining. Subsequently, the tissue sections were incubated with the corresponding secondary antibodies (Zsbio, Beijing, China) at 37 °C for 30 min and visualized using diaminobenzidine (DAB, Gene-Tech, South San Francisco, CA, USA). Tissue section images were acquired using an Aperio ScanScope (Leica microsystems, Wetzlar, Germany). The quantitative analysis of the stained areas was performed on three randomly selected regions within the sections using ImageJ software (ImageJ 1.x, NIH, Bethesda, MD, USA).

### 2.6. Measurement of MDA

The concentration of lipid peroxidation products was determined using a Malondialdehyde (MDA) Assay Kit (Sigma, St. Louis, MO, USA) following the manufacturer’s instructions. This assay employs the thiobarbituric acid (TBA) reaction to quantify MDA, and the absorbance of the MDA-TBA adduct was measured at 532 nm.

### 2.7. Iron Assay

Iron and ferrous ion (Fe^2+^) levels and ratios were assessed using an Iron Assay Kit (Abcam, Boston, MA, USA) per manufacturer guidelines. Fe^2+^ forms a stable color complex with a probe, measured at 593 nm. Fe^3+^ is reduced to Fe^2+^ for quantifying total iron (Fe^2+^/Fe^3+^).

### 2.8. Cell Viability

Cell proliferation post-treatment was evaluated using the Cell Counting Kit-8 (Biosharp, Hefei, China). The UM1 and CAL27 cell suspensions (200 µL, 2000 cells/well) were seeded into a 96-well plate. The cells were incubated at 37 °C with 5% CO_2_ in a humidified incubator with GW9662 (20 µM), T0070907 (20 µM), liproxstatin-1 (2 µM), or TCEP (1 mM). Drug treatments lasted for 24, 48, or 72 h. Each well was added with 20 µL of CCK-8 solution and incubated for 1.5 h. The absorbance at 450 nm was measured using an ultraviolet spectrophotometer.

### 2.9. Glucose Measurements

The glucose content in the culture media of the UM1 and CAL27 cells was determined using a glucose assay kit (Solarbio, Beijing, China) following the manufacturer’s protocol and was normalized to the total cell count in each sample.

### 2.10. NADPH Measurements

NADPH levels were measured using a double-antibody sandwich enzyme-linked immunosorbent assay (ELISA; Ruixin Biotech, Quanzhou, China). Quantification was based on cell counts, with absorbance read at 450 nm using a microplate reader. Concentrations were then calculated via a standard curve.

### 2.11. Western Blot Analysis

The cells were washed three times with sterile PBS and proteins were extracted using RIPA lysis buffer. Protein concentrations were determined using the Pierce™ BCA Protein Assay Kit (Thermo Fisher Scientific, Waltham, MA, USA). For reducing Western Blot, a protein loading buffer with a reducing agent was used, whereas the non-reducing Western Blot employed a buffer devoid of the reducing agent. The samples were heated at 95 °C for 10 min before SDS-PAGE electrophoresis and subsequent transfer onto PVDF membranes (Millipore, Bedford, MA, USA). After blocking with 5% skim milk for an hour, the membrane was incubated overnight at 4 °C with primary antibodies: anti-GLUT1 (Proteintech, Wuhan, China, 1:1000), anti-PPARγ (Abcam, Boston, MA, USA, 1:1000), anti-HMOX1 (Abcam, Boston, MA, USA, 1:2000), anti-GPX4 (Abcam, Boston, MA, USA, 1:2000), anti-p-NRF2 (Proteintech, Wuhan, China, 1:6000), anti-SLC7A11 (Abcam, Boston, MA, USA, 1:2000), anti-GAPDH (Abcam, Boston, MA, USA, 1:10,000), anti-Pan-Actin (Cell Signaling Technology, Danvers, MA, USA, 1:500), and anti-β-actin (Proteintech, Wuhan, China, 1:5000). The membrane was then incubated with HRP-conjugated anti-mouse or anti-rabbit secondary antibodies (ZSbio, Beijing, China, 1:10,000) for an hour. Following incubation with a chemiluminescent substrate, images were captured using the ImageReader AS-2000 (Fujifilm, Tokyo, Japan) and analyzed with the ImageJ software (ImageJ 1.x, NIH, Bethesda, MD, USA). Original figures can be found in Supplementary Materials.

### 2.12. Quantitative Real-Time PCR

Total RNA was extracted from each sample using the TRIzol reagent (GENSTONE BIOTECH, Beijing, China), and reverse transcription was performed using a PrimeScript RT Kit (Vazyme, Nanjing, China) according to the manufacturer’s protocol. The primers used for Q-PCR were as follows: Hmox1 forward: 5′-AAGCCGAGAATGCTGAGTTCA-3′, Hmox1 reverse: 5′-GCCGTGTAGATATGGTACAAGGA-3′; Slc7a11 forward: 5′-GCTTTGTCTTATGCTGAATTGG-3′, Slc7a11 reverse: 5′- TGCAGGGCGTATTATGAGGAG-3′; and Gapdh forward: 5′-AGGTCGGTGTGAACGGATTTG-3′, Gapdh reverse: 5′-GGGGTCGTTGATGGCAACA-3′. Quantitative PCR was performed using SYBR. A green system (Vazyme, Nanjing, China) was used, and the samples were run on an ABI 7300 Real-time PCR instrument (Thermo Fisher Scientific, Waltham, MA, USA).

### 2.13. Fluorescent Staining of Actin

The cells plated on slides were washed with PBS and fixed with 4% paraformaldehyde. After fixation, the cells were permeabilized with a permeabilization buffer (0.5% Triton X-100 in PBS) for 5 min. Actin filaments were stained with 100 nM Actin-stain 555 phalloidin (Cytoskeleton, Denver, CO, USA) for 30 min. The slides were then mounted with a DAPI-containing mounting medium (VECTASHIELD, Vector Labs, Burlingame, CA, USA) and examined under a fluorescence microscope.

### 2.14. Multiplexed Immunohistochemistry

The tissue sections were processed using the multiplexed immunohistochemistry (mIHC) kit (Akoya Biosciences, Marlborough, MA, USA) according to the manufacturer’s instructions. Primary antibodies used included CD8a (Cell Signaling Technology, Danvers, MA, USA, 1:400), CD11c (Cell Signaling Technology, Danvers, MA, USA, 1:400 dilution), and PANCK (Abcam, Boston, MA, USA, 1:2000). The cell nuclei were counterstained with ProLong™ Gold Antifade Mountant containing DAPI (Invitrogen, Carlsbad, CA, USA). Finally, the samples were meticulously examined under an immunofluorescence microscope to capture detailed fluorescent images.

### 2.15. CyTOF

The mouse OSCC tissues harvested from orthotopic tumor formation experiments were collected for analysis by time-of-flight mass spectrometry. The tumors from two mice were combined as one sample, preserved in a storage solution, and then submitted to PLTTECH (Hangzhou, China) for the comprehensive profiling of the immune characteristics in the OSCC tissues from different groups using CyTOF technology.

### 2.16. Data Processing

Data analysis was performed using the GraphPad Prism software (Version 8.0), and the data results are presented as mean ± standard deviation. The *p*-value was determined by unpaired Student’s *t*-test or ANOVA, with a corresponding sample size (*n*) ≥ 3. Statistical significance was determined at *p* < 0.05. “*” represents *p* < 0.05, “**” represents *p* < 0.01, and “***” represents *p* < 0.001.

## 3. Results

### 3.1. GW9662, an Antagonist of PPARγ, Induces Ferroptosis in OSCC Cells

**Figure 1:**
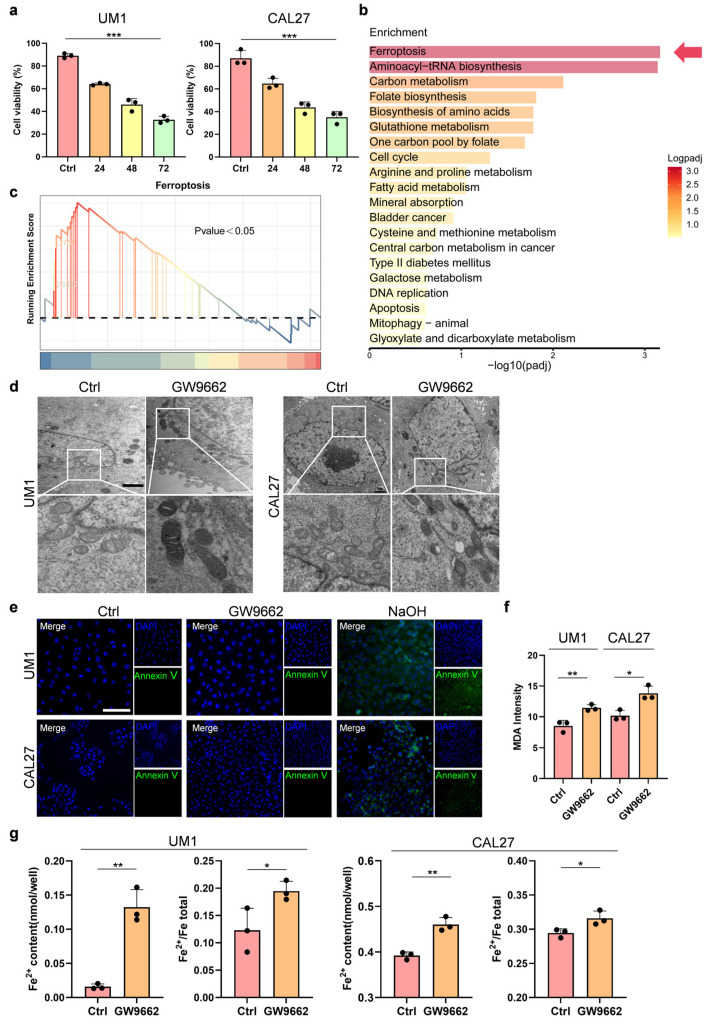
Inhibition of PPARγ promotes the ferroptosis phenotype in OSCC (**a**). The UM1 cells and CAL27 cells were treated without (Ctrl) or with a PPARγ receptor antagonist (GW9662, 20 µM) for 24 h, 48 h, or 72 h. Cell viability was quantified using a CCK-8 assay. (**b**). The OSCC cells were treated with or without a PPARγ receptor antagonist (GW9662, 20 µM) for 24 h, after which RNA sequencing was performed. KEGG enrichment analysis revealed the pathways most significantly affected by PPARγ inhibition among the DEGs. (**c**). GSEA revealed significant changes in ferroptosis pathway activity. (**d**). Transmission electron microscopy demonstrated that the OSCC cell mitochondria were altered following 24 h of treatment with a PPARγ receptor antagonist (scale bar, 1 µm). (**e**). TUNEL staining revealed no cell apoptosis in the PPARγ receptor antagonist-treated group (GW9662, 20 µM). NaOH (3% *w*/*v*) served as the positive control group (scale bar, 100 µm). (**f**). MDA content detection showed increased MDA levels in the OSCC cells treated with a PPARγ receptor antagonist (GW9662, 20 µM) compared to those in the control group (Ctrl). (**g**). Fe^2+^ concentration detection demonstrated elevated Fe^2+^ levels and proportions in the OSCC cells treated with a PPARγ receptor antagonist (GW9662, 20 µM) compared to those in the control group (Ctrl). The data are presented as the means ± SDs from three independent experiments. The asterisks indicate significant differences (Student’s *t*-tests, * *p* < 0.05, ** *p* < 0.01, and *** *p* < 0.001).

To explore the role of PPARγ in the growth of OSCC, we used GW9662, an antagonist of PPARγ [18], to treat the OSCC cells. The CCK-8 assays unveiled a significantly reduced survival rate in both OSCC cell lines within the GW9662-treated group compared to the Ctrl group (Figure 1a). Subsequently, we employed previously acquired RNA-seq data [8] to assess the differentially expressed genes (DEGs) and related signaling pathways in the OSCC cells treated with or without GW9662. As a result, the expression of multiple ferroptosis-related genes increased, and ferroptosis-related pathways were enriched in the GW9662 treatment group (Figure 1b,c). We then used transmission electron microscopy (TEM) to observe the changes in subcellular structure in the OSCC cells treated with or without GW9662 and found that the number of mitochondria in the cells decreased and that the number of mitochondrial cristae was reduced or even absent in the GW9662-treated cells (Figure 1d). Subsequently, to exclude the occurrence of other programmed deaths, we used an apoptosis detection kit to verify that the OSCC cells did not undergo apoptosis after the GW9662 treatment, and the results were consistent with our expectations (Figure 1e). Moreover, we found that the lipid peroxide malondialdehyde (MDA) content of the OSCC cells increased after the GW9662 treatment (Figure 1f). In addition, the content and proportion of ferrous ions in the OSCC cells increased after the GW9662 treatment (Figure 1g). In conclusion, these results strongly suggest that the inhibition of PPARγ can promote ferroptosis in OSCC cells.

### 3.2. OSCC Cells Undergo Disulfidptosis in Addition to Ferroptosis after Treatment with an Antagonist of PPARγ

**Figure 2:**
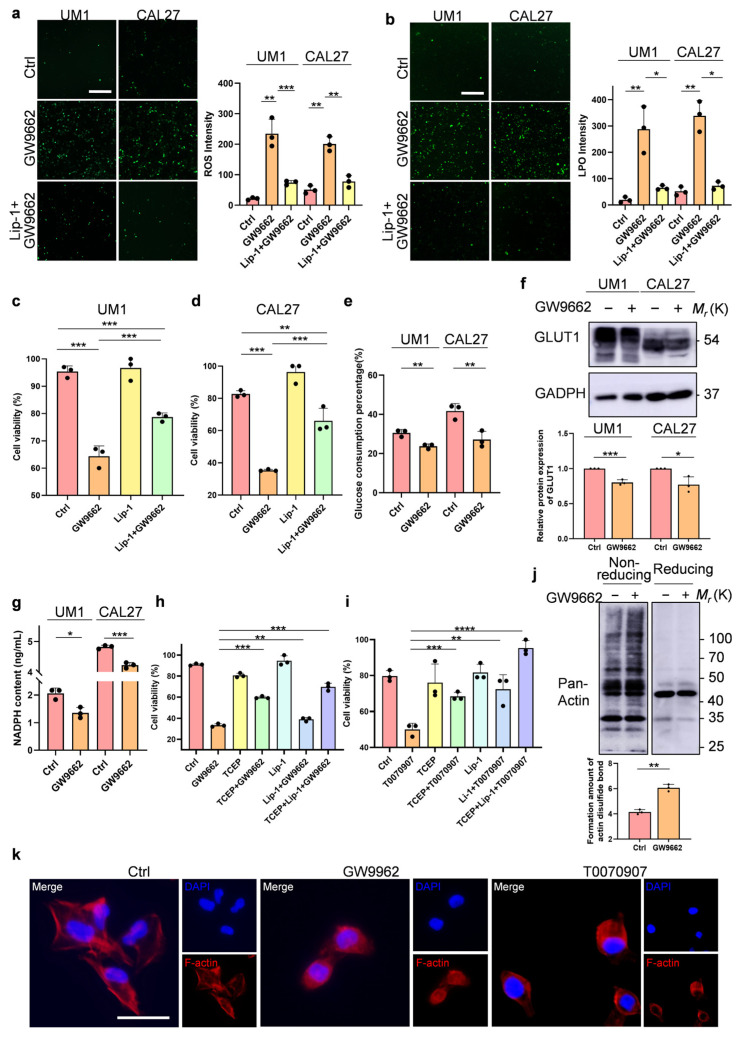
Impact of PPARγ inhibition on ferroptosis and disulfidoptosis in the OSCC cells (**a**,**b**). Fluorescence images depicting intracellular ROS and LPO fluorescence intensity in the OSCC cells following various treatments: control, GW9662 (20 µM), and liproxstatin (2 µM) combined with GW9662 (20 µM) (scale bar: 100 µm). (**c**,**d**). The CCK-8 assay illustrating the rescue effect of the ferroptosis inhibitor liproxstatin (2 µM) on the decrease in cell viability induced by the PPARγ receptor antagonist GW9662 (20 µM). (**e**). Glucose content detection showing the impact of a PPARγ receptor antagonist (GW9662, 20 µM) on glucose consumption in the OSCC cells. (**f**). The Western blotting analysis showing the inhibitory effect of a PPARγ receptor antagonist (GW9662, 20 µM) on the expression of the GLUT1 protein. (**g**). NADPH content detection showed that the PPARγ receptor antagonist (GW9662, 20 µM) reduced the NADPH content in the OSCC cells. (**h**,**i**). The CCK-8 assays demonstrated the synergistic effect of a ferroptosis inhibitor (liproxstatin-1, 2 µM) and a disulfidptosis inhibitor (TCEP, 1 mM) on rescuing cell death induced by PPARγ receptor antagonists (GW9662, T0070907, 20 µM). (**j**). The non-reducing and reducing Western blot analysis of indicated actin in the UM1 cells treated with or without GW9662 (20 µM) for 24 h. (**k**). The fluorescence staining of F-actin and DAPI staining of the OSCC cells cultured in GW9662 (20 µM) for 24 h (scale bar: 1 µm). Lip-1 represents liproxstatin-1. The data are presented as the means ± SDs from three independent experiments. The asterisks indicate significant differences (Student’s *t*-tests, * *p* < 0.05, ** *p* < 0.01, and *** *p* < 0.001).

To verify the occurrence of ferroptosis, we simultaneously used GW9662 and/or a ferroptosis inhibitor to observe the formation of reactive oxygen species (ROS) and lipid peroxides (LPOs) under a fluorescence microscope. We found that the concentration of ROS produced by the OSCC cells increased significantly after the GW9662 treatment and was inhibited by the addition of ferroptosis inhibitors (Figure 2a). Consistent with these findings, the level of LPO produced by the OSCC cells increased significantly after the GW9662 treatment, while in the presence of ferroptosis inhibitors, the concentration of LPO decreased markedly (Figure 2b). As a highly selective activator of PPARγ, rosiglitazone can effectively restore the death of the OSCC cells induced by GW9662 (Supplemental Figure S1). This result reinforces our hypothesis that PPARγ is a key target in regulating OSCC cell death. Surprisingly, the CCK-8 assays revealed that the ferroptosis inhibitor liproxstatin-1 partially rescued cell death caused by GW9662 (Figure 2c,d).

To unravel the reason behind the incomplete rescue of cell death by ferroptosis inhibitors following PPARγ inhibition, we explored disulfidptosis, a new type of cell death; under glucose starvation, the supply of NADPH is insufficient, and the high expression of SLC7A11 in cancer cells induces abnormal accumulation of disulfides, leading to cell death [17]. Our transcriptome sequencing data indicated a significant increase in the expression of SLC7A11 following the treatment with GW9662, and PPARγ has been reported to be related to glucose uptake and transport [19,20,21,22]. Based on the above findings, we explored whether disulfidptosis occurs when PPARγ is inhibited. First, we measured the glucose consumption of the OSCC cells in a complete culture medium within 48 h and found that the glucose consumption decreased after PPARγ was inhibited (Figure 2e). Considering that the inhibitors of the glucose transporter GLUT1 can effectively inhibit cellular glucose uptake and thereby induce disulfidptosis in cells with high SLC7A11 expression [15,17], we investigated the protein expression of GLUT1 and expectedly observed a substantial reduction in GLUT1 expression after PPARγ was inhibited (Figure 2f). Next, we detected the content of NADPH enzyme in the cells using ELISA and found that the content of NADPH enzyme was markedly reduced after the OSCC cells were treated with both antagonists of PPARγ (Figure 2g). Notably, the disulfidptosis inhibitor TCEP also noticeably rescued cell death caused by GW9662 or the other PPARγ receptor antagonist T0070907, and the rescue effect on cell death was the greatest when both TCEP and liprostatin-1 were present at the same time (Figure 2h,i and Figure S2a,b). The formation of disulfide bonds between actin molecules leads to protein aggregation or cross-linking, resulting in complexes of varying sizes. Under non-reducing conditions, this diversifies their migration rates in the gel, consequently forming broad smearing bands. Hence, leveraging insights from prior scholarly investigations [17], we undertook a non-reducing Western blot assay to ascertain the presence of disulfide bonds in actin. Following a 24 h exposure of the UM1 and CAL27 cells to a PPARγ antagonist, a notable retardation in the electrophoretic migration of actin was observed (Figure 2j and Figure S2c). Subsequently, after treatment with both antagonists of PPARγ, the cell cytoskeleton shrank significantly in the OSCC cells (Figure 2k and Figure S2d). These data clearly demonstrate that the OSCC cells undergo both ferroptosis and disulfidptosis after PPARγ inhibition.

### 3.3. HMOX1 and SLC7A11 Are Upregulated to Promote Ferroptosis and Disulfidptosis, Respectively, through the Inhibition of PPARγ

**Figure 3:**
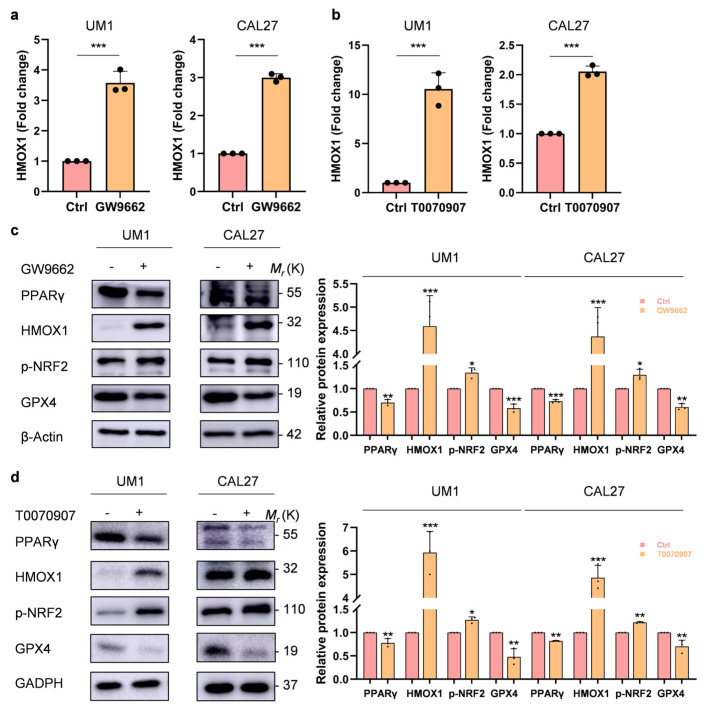

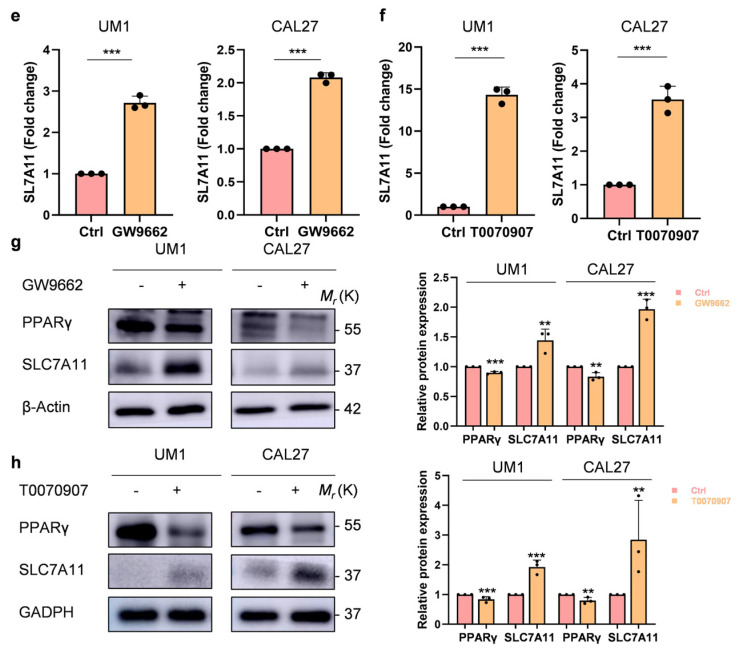
Regulation of ferroptosis by upregulating the HMOX1/NRF2 pathway and disulfidptosis by upregulating SLC7A11 through PPARγ inhibition in OSCC (**a**,**b**). qRT–PCR analysis revealed increased mRNA expression levels of HMOX1 following treatment of the OSCC cells with a PPARγ receptor antagonist (GW9662, T0070907, 20 µM) for 24 h. (**c**,**d**). The Western blot analysis of the protein expression levels of PPARγ, HMOX1, p-NRF2, and GPX4 in the OSCC cells treated with both PPARγ receptor antagonists (GW9662, T0070907, 20 µM) for 24 h. (**e**,**f**). qRT–PCR demonstrated elevated SLC7A11 mRNA expression in the OSCC cells upon treatment with a PPARγ receptor antagonist (GW9662, T0070907, 20 µM) for 24 h. (**g**,**h**). Western blot analysis illustrated the protein expression levels of SLC7A11 in the OSCC cells treated with a PPARγ receptor antagonist (GW9662, T0070907, 20 µM) for 24 h. The data are presented as the means ± SDs from three independent experiments. The asterisks indicate significant differences (Student’s *t*-tests, * *p* < 0.05, ** *p* < 0.01, and *** *p* < 0.001).

Our transcriptome sequencing revealed changes in multiple ferroptosis-related genes, among which HMOX1 was highlighted. HMOX1 plays a pivotal role in heme degradation, breaking down hemoglobin into carbon monoxide, free iron, and bilirubin [23,24]. qPCR confirmed that the expression of HMOX1 was notably upregulated in the OSCC cells treated with both the PPARγ inhibitors GW9662 and T0070907 (Figure 3a,b). After PPARγ inhibition, the expression of HMOX1 and its transcription factor p-NRF2 was significantly increased, and the expression of the ferroptosis core regulator glutathione peroxidase 4 (GPX4) was significantly decreased (Figure 3c,d). Moreover, the mRNA expression level of SLC7A11 was upregulated after treatment with GW9662 or T0070907 in the OSCC cells, as detected by qPCR (Figure 3e,f). The protein expression level of SLC7A11 was consistent with its mRNA level (Figure 3g,h). These results indicate that reduced PPARγ activity can upregulate the expression of HMOX1 and SLC7A11, which promote ferroptosis and disulfidptosis, respectively.

### 3.4. Reduced PPARγ Activity Could Promote the Anticancer Effects of OSCC by Inhibiting Ferroptosis and Disulfidptosis

**Figure 4:**
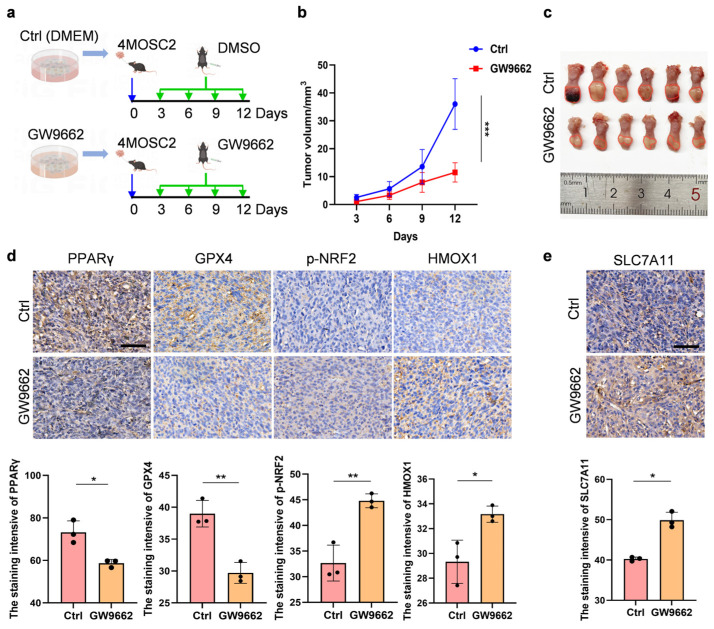
Impact of PPARγ inhibition on OSCC tumor development via ferroptosis and disulfidptosis promotion (**a**). The schematic diagram of the in vivo studies. The experimental group received GW9662 at 2.5 mg/kg, while the control group received DMSO injection once every three days. *n* = 6 mice/group. (**b**). Changes in tumor volume in the mice. (**c**). The schematic diagram of a mouse tongue containing OSCC tumors. (**d**,**e**). Immunohistochemistry was performed on OSCC tissue to assess the expression of PPARγ, GPX4, p-NRF2, HMOX1, and SLC7A11. The calculation of the positive area and intensity was also conducted (scale bar: 50 µm). The data are presented as the means ± SDs from three independent experiments. The asterisks indicate significant differences (Student’s *t*-tests, * *p* < 0.05, ** *p* < 0.01, and *** *p* < 0.001).

To explore whether the antagonists of PPARγ have anticancer effects on OSCC, we generated orthotopic OSCC tumors in the tongues of mice and treated the mice with GW9662 (Figure 4a). The tumors derived from the group treated with GW9662 were smaller than those derived from the control group, and the tumor volumes were significantly reduced (Figure 4b,c and Figure S3). To determine whether ferroptosis and disulfidptosis contribute to anticancer effects in GW9662-treated mouse tumor models, the expression levels of PPARγ, HMOX1, GPX4, p-NRF2, and SLC7A11 in tumor tissues were assessed via IHC staining. As expected, PPARγ and GPX4 levels were lower in the GW9662-treated tumor tissues than in the control tumors. HMOX1, p-NRF2, and SLC7A11 were expressed at higher levels in the GW9662-treated tumor tissues than in the control tumor tissues (Figure 4d,e). In conclusion, reducing PPARγ activity could inhibit the growth and development of OSCC by promoting ferroptosis and disulfidptosis.

### 3.5. The Number of DCs and CD8^+^ T Cells Increased in OSCC after PPARγ Activity Was Reduced

**Figure 5:**
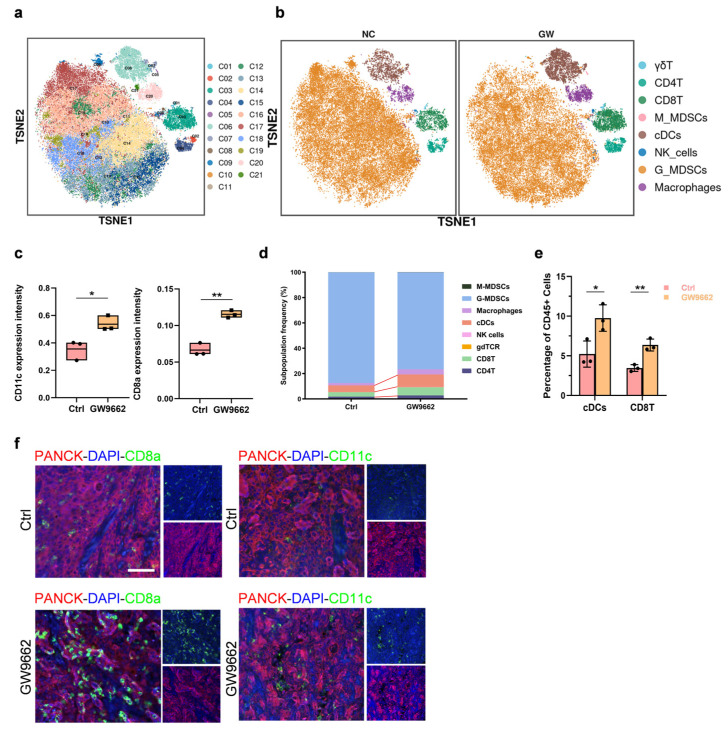
PPARγ inhibition recruited more cDCs and CD8^+^ T cells to the OSCC tumor microenvironment (**a**). Two-dimensional t-SNE plot of the merged CyTOF data from the OSCC tissues of both the CTRL group and the GW9662 group. (*n* = 6 mice/group). (**b**). The ViSNE analysis of the stained and labeled immune cells in the two groups of tissues. (**c**). The expression levels of CD11c and CD8a in the two groups. (*n* = 6 mice/group). (**d**). The statistical analysis of the changes in the proportions of cDCs and CD8^+^ T cells in the two groups. (**e**). The composition of the CD45^+^ compartment shows the average proportion of major immune lineages in the OSCC tissues of the CTRL group and the GW9662 group. (**f**). mIHC depicted increased numbers of cDCs and CD8^+^ T cells in the OSCC tissues from the GW9662 group (scale bar: 50 µm). The data are presented as the means ± SDs from three independent experiments. The asterisks indicate significant differences (Student’s *t*-tests, * *p* < 0.05, ** *p* < 0.01).

Since ferroptosis and other modes of cell demise have the potential to reshape the tumor immune microenvironment, they can significantly influence tumor development [25,26]. Subsequently, we analyzed variations in immune cell subsets within the tumor tissues between the GW9662-treated group and the control group using single-cell mass cytometry (CyTOF) on the entire OSCC cohort from both groups after 12 days. Employing a panel encompassing 42 immune protein markers (Supplemental Table S1), we delineated the primary immune cell types and charted the intricate landscapes of various immune checkpoint genes. Using a single-cell immune atlas, we examined the healthy and infected OSCC tissues and identified several clusters, such as CD4^+^ T cells, CD8^+^ T cells, conventional dendritic cells (cDCs), natural killer (NK) cells, and macrophages. This was achieved through the visualization of well-characterized cell markers on two-dimensional (2D) t-distributed stochastic neighbor embedding (t-SNE) plots. (Figure 5a,b). We found that the expression of CD11c and CD8a was markedly greater in the GW9662-treated tumor tissues than in control tumor tissues (Figure 5c), and the quantity of cDCs and CD8^+^ T cells was notably greater in the GW9662-treated tumor tissues than in the control tissues (Figure 5d,e). Through multiplex immunohistochemistry (mIHC), we confirmed that the number of cDCs and CD8^+^ T cells in the OSCC tumors treated with the PPARγ inhibitor was significantly greater than that in the control group (Figure 5f). The above findings illustrated that upon PPARγ inhibition in OSCC, tumor cells could undergo ferroptosis and disulfidptosis, thereby promoting the recruitment of cDCs and CD8^+^ T cells to inhibit the occurrence of OSCC tumors.

## 4. Discussion

OSCC is the main type of head and neck squamous cell carcinoma and has a high degree of malignancy. Although surgical resection supplemented by radiotherapy, chemotherapy, and targeted therapy are the standard treatments for OSCC, the 5-year overall survival rate remains considerably low. The main reasons are the complexity and uncertainty of the OSCC microenvironment and the lack of reliable therapeutic targets. Therefore, there is an urgent need to discover valuable targets for the treatment of OSCC. Previous research on PPARγ has focused mainly on the role of PPARγ in regulating lipid metabolism, glucose metabolism, etc. [27,28]. With further research, the role of PPARγ in tumors has become increasingly obvious. However, its role in tumors remains controversial. Here, we found that inhibiting PPARγ in OSCC can promote ferroptosis by upregulating HMOX1 and promoting disulfidptosis through the upregulation of SLC7A11 and the subsequent recruitment of additional cDCs and CD8^+^ T cells to inhibit tumor development.

GW9662, as a potent PPARγ antagonist, acts by covalently binding to the ligand-binding domain of the receptor, effectively blocking the activity of other agonists, rather than inducing large-scale receptor conformational remodeling. The binding of GW9662 does not significantly alter the interaction balance between PPARγ and coactivators or corepressors, thereby exhibiting a transcriptionally neutral effect. T0070907, an analog of GW9662, differs from it by a carbon-to-nitrogen substitution. When bound to PPARγ, T0070907 exhibits two long-lived conformational states, one of which is similar to the binding state of GW9662 [29,30]. These changes may not directly inhibit all receptor functions but subtly adjust the transcriptional program by affecting its interaction with specific coregulatory factors, resulting in transcriptionally neutral or selective effects on certain gene expressions. This fine-tuning of conformation can preserve or alter the partial activity of the receptor, affecting the delicate regulation of downstream signaling pathways. In contrast, silencing PPARγ using RNA interference technology (such as shRNA or siRNA) directly reduces the total amount of this receptor in cells. This strategy not only prevents the activating effect of agonists but may also comprehensively affect all PPARγ-dependent biological processes, including those basic activities that can still be maintained in an antagonist state. PPARγ plays a crucial role in physiological and pathological processes, and its activation or inhibition can profoundly affect the occurrence and development of various tumors. Given the central position of PPARγ in regulating multiple cellular functions, small changes in its active state may cause significant differences in physiological or pathological effects. The distinction between structural changes and overall expression reduction suggests that we need to carefully consider the specificity and potential systemic effects of intervention strategies when designing therapies targeting PPARγ.

Currently, PPARγ receptor antagonists have been found in various cancer studies to inactivate PPARγ to regulate tumor occurrence and development [31,32,33]. Previous studies have reported that PPARγ inhibitors may contribute to ferroptosis in a variety of diseases [34,35]. We also found that the PPARγ inhibitors GW9662 and T0070907 promoted ferroptosis in the OSCC cells, leading to cell death. However, ferroptosis inhibitors cannot completely rescue cell death caused by the PPARγ inhibitors. We found that inhibiting PPARγ not only caused ferroptosis in the OSCC cells but also caused disulfidptosis. The identification and characterization of ferroptosis and disulfidptosis not only enhance the fundamental understanding of cellular homeostasis but also provide crucial insights for the treatment of various diseases, especially cancer. SLC7A11 is not only a potent target of ferroptosis but is also considered an adaptive resistance barrier molecule that promotes cancer therapy, and it also plays a crucial regulatory role in disulfidptosis. However, current research often treats disulfidptosis and ferroptosis as distinct processes, and investigators have discovered that disulfidptosis actually excludes the occurrence of ferroptosis [17]. It is currently unclear whether the relationships between them are mutually exclusive or whether they may coexist. Notably, in our research, the inhibition of PPARγ in OSCC was shown to induce both ferroptosis and disulfidptosis simultaneously. Here, we propose for the first time that targeting PPARγ can link these two death pathways to inhibit tumor cell proliferation.

Cell death can lead to the release of immunogenic substances within cells. The signaling molecules released by dying cells can activate relevant receptors on the surface of immune cells, prompting these cells to enter a more active state and enhancing their aggressiveness against tumor cells. Stimulating immunogenic cell death in tumor cells not only directly kills tumor cells but also further eliminates residual or metastatic lesions, preventing tumor recurrence [36]. The combinations of multiple cell death pathways can generate synergistic effects, including reducing tumor-related antiapoptotic signals, lowering the levels of immune suppressive factors, and enhancing the killing efficacy against tumor cells. This comprehensive approach can avoid cellular escape mechanisms by simultaneously targeting multiple vulnerabilities, thereby increasing the overall effectiveness of treatment. Ferroptosis can alter the tumor microenvironment, for example, by secreting inflammatory factors to activate immune-related cells, such as CD4^+^ T cells and CD8^+^ T cells, or by producing metabolic molecules such as phospholipid peroxides, thereby influencing immune cells [37]. Although research on disulfidptosis is currently limited, some researchers have found that genes associated with disulfidptosis are linked to various immune cells. It holds significant potential in influencing the tumor microenvironment [38,39]. Our data confirmed that the inhibition of PPARγ in OSCC simultaneously induces ferroptosis and disulfidptosis. This leads to the recruitment of more cDCs and CD8^+^ T cells to the tumor microenvironment, effectively suppressing tumor progression. Targeting tumor cell death induced by the inhibition of PPARγ combined with immunotherapy may yield favorable outcomes in the treatment of OSCC or other cancers.

In this study, we observed a significant reduction in the viability of OSCC cells in vitro upon treatment with PPARγ inhibitors. This occurs by upregulating HMOX1 to promote ferroptosis and concurrently upregulating SLC7A11 to induce disulfidptosis. Treatment with PPARγ inhibitors in an in vitro OSCC model resulted in a substantial increase in the number and proportion of cDCs and CD8^+^ T cells in tumor tissue, leading to a significant inhibition of tumor growth. In light of these discoveries, the role of PPARγ in ferroptosis and disulfidptosis may be explored in future research to improve the clinical treatment of OSCC and other diseases.

## Data Availability

The raw data supporting the conclusions of this article will be made available by the authors upon request.

## Supplementary Materials

The following supporting information can be downloaded at: https://www.mdpi.com/article/10.3390/biom14050596/s1, Figure S1: Rosiglitazone rescues cell death induced by GW9662; Figure S2: Effects of inhibiting PPARγ on ferroptosis and disulfidptosis in CAL27; Figure S3: The alterations in the protein levels of PPARγ in mouse OSCC. Table S1: Antibodies used for CyTOF staining.
