# Supplementary material for: PPARγ Antagonists Exhibit Antitumor Effects by Regulating Ferroptosis and Disulfidptosis
Source: Biomolecules. 2024 May 18;14(5):596. doi: 10.3390/biom14050596 (PMC11118805; doi:10.3390/biom14050596)
Supplement: Supplementary file 1 [file biomolecules-14-00596-s001.zip › biomolecules-2959002-supplementary.pdf]

PPARγ antagonists exhibit antitumor effects by regulating ferroptosis and disulfidptosis

Shiyu Zhang[1 †], Ying Wang[1 †], Junjie Gu[1], Yang Yang[1], Jing Liang[1], Yimei Wang[1], Ning Ji[1], Ming Liu[1], Yingxin Zhang[1], Silu Sun[1], Qianming Chen[1], Jing Li[1*]

[1]State Key Laboratory of Oral Diseases, National Clinical Research Center for Oral Diseases, Research Unit of Oral Carcinogenesis and Management, Chinese Academy of Medical Sciences, West China Hospital of Stomatology, Sichuan University, Chengdu, China

✉e-mail: lijing1984@scu.edu.cn (J.L.)

This file includes:

- **Figure S1, S2, S3**
- **Table S1**

Supplemental Figures and Figure Legends

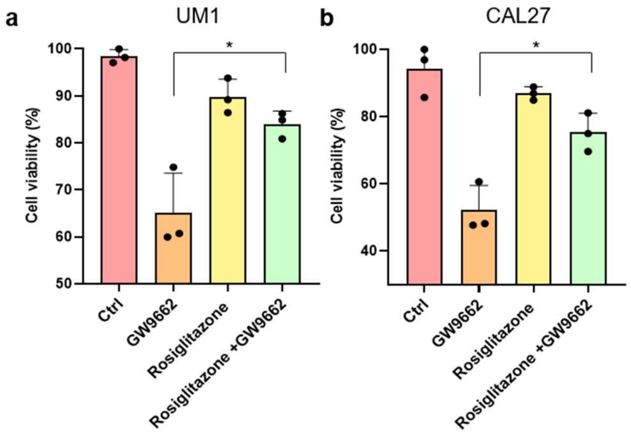

Supplemental Fig S1. Rosiglitazone rescues cell death induced by GW9662. a-b. CCK-8 assay demonstrates that the PPARγ agonist (rosiglitazone, 10 μM) significantly rescues OSCC cell death induced by the PPARγ receptor antagonist (GW9662, 20 μM). Data represent mean values ± SD from three independent experiments. The asterisks indicate significant differences (Student's t tests, *$P < 0.05$, **$P < 0.01$, ***$P < 0.001$).

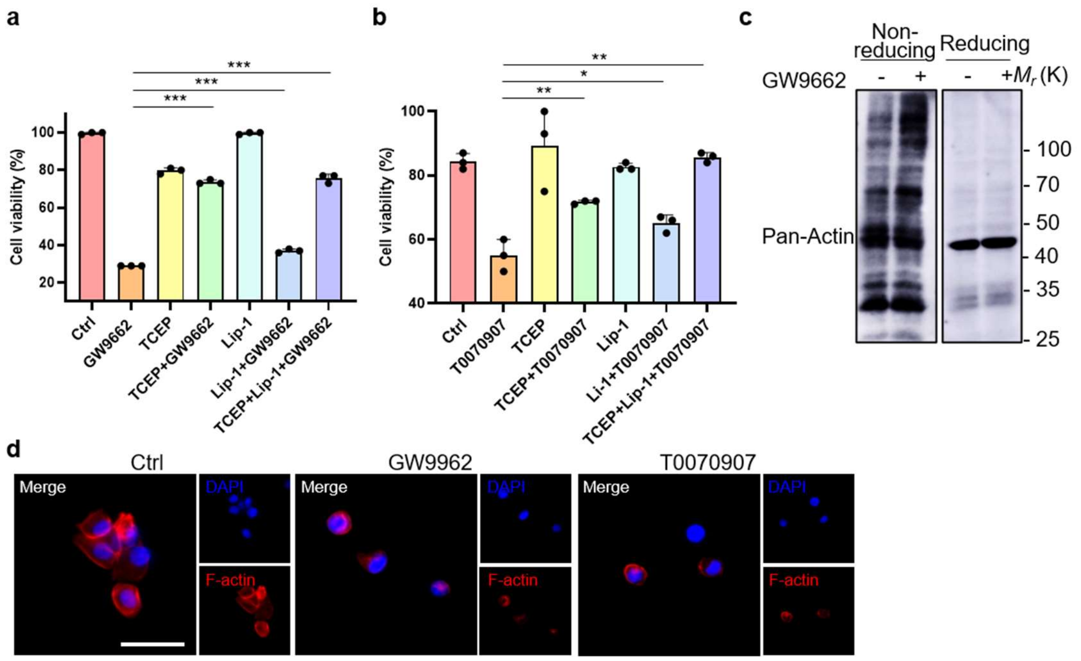

Supplemental Fig S2. Effects of inhibiting PPARγ on ferroptosis and disulfidptosis in CAL27. a-b. CCK-8 assay demonstrated the synergistic effect of ferroptosis inhibitor (Liproxstatin-1, 2 μM) and disulfidptosis inhibitor (TCEP, 1 mM) in rescuing CAL27 cell death induced by PPARγ receptor antagonists (GW9662, T0070907, 20 μM). **c**. Non-reducing and reducing western blot analysis of indicated actin in CAL27 cells treated with or without GW9662(20 μM) for 24 hours. **d.** Fluorescence staining of F-actin and DAPI in CAL27 cells cultured in GW9662 (20 μM) for 24 hours (Scale bar: 1 μm). Lip-1 represents Liproxstatin-1. Data represent mean values ± SD from three independent experiments. The asterisks indicate significant differences (Student's t tests, *P < 0.05, **P < 0.01, ***P < 0.001).

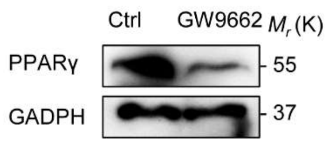

Supplemental Fig S3. The alterations in the protein levels of PPARγ in mouse OSCC. Western blot detected the protein expression levels of PPARγ in mouse OSCC tumors treated with GW9662.

Table S1: Antibodies used for CyTOF staining

List	Label	Marker	Clone
1	89Y	CD45	30-F11
2	115ln	CD3ε	145-2C11
3	139La	Ki-67	SolA15
4	141Pr	CD95(Fas)	SA367H8
5	142Nd	CD11c	N418
6	143Nd	CD366(Tim-3)	RMT3-23
7	144Nd	CX3CR1	SA011F11
8	145Nd	T-bet	4B10
9	146Nd	CD27	LG.3A10
10	147Sm	CD161(NK-1.1)	PK136
11	148Nd	Ly-6C	HK1.4
12	149Sm	CD172a(SIRPα)	P84
13	150Nd	CD25	3C7
14	151Eu	CD44	IM7
15	152Sm	CD19	6D5
16	153Eu	CD274(PD-L1)	10F.9G2
17	154Sm	CD194(CCR4)	2G12
18	155Gd	CD223(LAG-3)	C9B7 W
19	156Gd	FOXP3	FJK-16s
20	157Gd	CD39	5F2
21	158Gd	VISTA(PD-1H)	MIH63
22	159Tb	F4/80	Cl:A3-1
23	160Gd	CD62L	MEL-14
24	161Dy	TIGIT(VSTM3)	2190A
25	162Dy	CD206(MMR)	C068C2
26	163Dy	Ly-6G	1A8
27	164Dy	CD103	2E7
28	165Ho	CD278(ICOS)	C398.4A
29	166Er	CD192(CCR2)	475301
30	167Er	CD184(CXCR4)	L276F12
31	168Er	CD49b(pan-NK cells)	DX5
32	169Tm	CD127(IL-7Rα)	A7R34
33	170Er	iNOS	CXNFT
34	171Yb	CD69	H1.2F3
35	172Yb	CD279(PD-1)	29F.1A12
36	173Yb	Granzyme B Recombinant	QA16A02
37	174Yb	CD196(CCR6)	29-2L17
38	175Lu	TCR β chain	H57-597
39	176Yb	MHC II(I-A/I-E)	M5/114.15.2
40	197Au	CD4	RM4-5

41	198Pt	CD8a	53-6.7
42	209Bi	CD11b	M1/70
